# Dr. Sylvester Emery Strong

**Published:** 1913-05

**Authors:** 


Dr. Sylvester Emery Strong, N. Y. University, 1862, died at Saratoga Springs, March 17, aged 75. He was surgeon and acting medical director of the Army of the Cumberland during the Civil War. In 1864 he established a sanatorium bearing his name and continued in this work throughout his life.

